# Nonclassical antagonism between human lysozyme and AMPs against *Pseudomonas aeruginosa*

**DOI:** 10.1002/2211-5463.13094

**Published:** 2021-02-05

**Authors:** Ian Blumenthal, Lydia R. Davis, Chet M. Berman, Karl E. Griswold

**Affiliations:** ^1^ Thayer School of Engineering Dartmouth, Hanover NH USA; ^2^ Lyticon LLC Lebanon NH USA; ^3^ Present address: University of Washington Seattle WA USA

**Keywords:** lysozyme, antimicrobial peptide, synergy, antagonism, antibiotic‐resistant, *Pseudomonas aeruginosa*

## Abstract

Combinations of human lysozyme (hLYS) and antimicrobial peptides (AMPs) are known to exhibit either additive or synergistic activity, and as a result, they have therapeutic potential for persistent and antibiotic‐resistant infections. We examined hLYS activity against *Pseudomonas aeruginosa* when combined with six different AMPs. In contrast to prior reports, we discovered that some therapeutically relevant AMPs manifest striking antagonistic interactions with hLYS across particular concentration ranges. We further found that the synthetic AMP Tet009 can inhibit hLYS‐mediated bacterial lysis. To the best of our knowledge, these results represent the first observations of antagonism between hLYS and AMPs, and they advise that future development of lytic enzyme and AMP combination therapies considers the potential for antagonistic interactions.

## Graphical abstract

Combinations of human lysozyme (hLYS) and antimicrobial peptides (AMPs) typically manifest synergistic activity against bacterial pathogens, and as a result, they have therapeutic potential for persistent and antibiotic‐resistant infections. Here, for the first time, we show that combinations of hLYS and some synthetic AMPs exhibit nonclassical antagonistic activity, suggesting the need to consider detrimental hLYS interactions during AMP drug development.

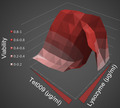

**Abbreviations**
hLYS: human lysozyme
AMP: antimicrobial peptide
PTG1: protegrin 1 AMP
hBD3: human beta defensin 3 AMP
DT: dextrose/tris screening buffer
FIC: fractional inhibitory concentration
FICI: fractional inhibitory concentration index
*V*_max_: maximum reaction velocity

Antibiotic‐resistant pathogens such as *Pseudomonas aeruginosa* pose a serious and growing threat to human health [1]. *P. aeruginosa* is an opportunistic Gram‐negative bacterium that can infect numerous tissues and organs [2, 3]. It is the dominant pathogen associated with cystic fibrosis, a genetic disorder in which patients suffer from treatment‐refractory lung infections that typically lead to respiratory failure [4]. More generally, *P. aeruginosa* encodes a diverse array of countermeasures against conventional antibacterial chemotherapies, and it can rapidly develop resistance to standard treatment regimens [5, 6]. Thus, there is an urgent need to develop novel antibacterial agents to more effectively combat *P. aeruginosa* [7, 8].

Bacteriolytic enzymes, such as human lysozyme (hLYS), have drawn long‐standing interest as potential treatments for antibiotic‐resistant bacteria. These agents function by effecting bacterial lysis via catalytic hydrolysis of cell wall peptidoglycan, and they are at the forefront of next‐generation antibiotic development [9]. While most studies of bacteriolytic enzymes focus on Gram‐positive pathogens, there is growing interest in anti‐Gram‐negative enzymes [10, 11], including hLYS [12, 13, 14, 15]. Importantly, however, the outer membrane of Gram‐negative bacteria shields the underlying peptidoglycan from ready access by lytic enzymes, and enzyme‐mediated lysis of Gram‐negative bacteria therefore necessitates penetrating this outer membrane barrier.

Antimicrobial peptides (AMPs) are short peptides with diverse secondary structure that, along with hLYS, comprise important molecular components of animal innate immunity. AMPs represent another attractive source of antibacterial agents [16, 17, 18], due in part to their unique mode of action: membrane disruption. A well‐established body of literature has found antibacterial synergy between hLYS and most AMPs (Table S1), with the remainder of tested combinations exhibiting additive interactions. Thus, combinations of hLYS and AMPs offer a promising new paradigm for treating antibiotic‐resistant infections, particularly those caused by Gram‐negative pathogens. Here, we evaluated a small panel of six AMPs combined with hLYS, and in contrast to all prior reports, we found that at least two of these combinations exhibited striking antagonistic activity across specific concentration ranges.

## Materials and methods

### Bacterial strains and antimicrobial agents

The mucoid bioluminescent *P. aeruginosa* strain Xen05 was purchased from Caliper life Sciences, Inc. The nonmucoid bioluminescent strain H1001 was the kind gift of Robert Hancock (University of British Columbia, Vancouver, BC, Canada). Both strains were stored as glycerol stocks at −80 °C. Recombinant hLYS and freeze‐dried *Micrococcus luteus* were purchased from Sigma Millipore (Burlington, MA, USA). Protegrin 1 AMP (PTG1) and HB71 peptides (> 95% purity) were obtained from Peptide 2.0. LL‐37, Melittin, and Tet009 peptides (> 95% purity) were purchased from GenScript (Piscataway, NJ, USA). Human beta defensin 3 AMP (hBD3) peptide was purchased from AnaSpec. Peptide stock solutions were stored at −20 °C. All other reagents and materials were purchased from Fisher Scientific (Waltham, MA, USA).

### Antibacterial EC_50_ assay

We analyzed the dose–response potency of each AMP against both strains via a well‐validated luminescence assay [19]. Hundred microlitre of exponential phase cells (5 × 10^6^ cells/mL) in dextrose/tris (DT) buffer (20 mm dextrose, 100 mm Tris, pH 7.4) were incubated with a 1 : 2 dilution series of each AMP in white, flat‐bottomed, 96‐well plates at 37 °C for 4 h. Culture luminescence was then quantified by imaging with a CCD camera (Bio‐Rad ChemiDoc XRS System, Hercules, CA, USA). Measurements were normalized to the no‐treatment controls on the same plate, and potency was quantified by a 3‐parameter logistic regression of luminescence versus AMP concentration, yielding EC_50_ values (Fig. S1). Importantly, the luminescence of these *P. aeruginosa* strains is known to correlate with bacterial viability [20]. Assays were done in technical triplicate or quadruplicate and repeated at least twice.

### Luminescence checkerboard assay

Checkerboard, or 2‐D MIC, assays were performed analogous to the single agent dose–response studies above, except that 1 : 2 serial dilutions of hLYS were made across the rows of a 96‐well plate, and 1 : 2 serial dilutions of the AMP were made down the columns of the same plate, such that each well contained a unique concentration mixture of the two agents. Controls without treatment (growth control) and without bacteria (sterility control) were included on all plates. Assays were repeated as three independent trials (biological replicates), and results are the average of all measurements.

To quantify antibacterial interactions, fractional inhibitory concentrations (FIC) and FIC index values (FICI) were calculated as described elsewhere [21], though in this case using EC_50_ values in place of conventional growth/no growth visual observations. Briefly, the FIC for one agent is the ratio of its EC_50_ value as a standalone treatment (denominator) and the concentration of that agent yielding a 50% luminescence reduction in the presence of a fixed concentration of a second agent (numerator). The corresponding FIC values for the two agents in each row and column, respectively, of a checkerboard plate may be summed, and the lowest FIC sum on the plate is designated the FICI.

### Lysozyme kinetic assays with *Micrococcus luteus*

hLYS lysis rates for *M. luteus* were measured using an adaptation of our previously reported methods [22]. Briefly, lytic rates were quantified in 96‐well plates by tracking *M. luteus* turbidity reduction as a function of time. Reactions, run in DT buffer, contained 200 ng·mL^−1^ hLYS and freeze‐dried *M. luteus* bacterial substrate at concentrations ranging from 50 to 500 µg·mL^−1^. Initial reaction rates were determined from slopes of the linear portions of time course data, and pseudo‐Michaelis–Menten kinetics (i.e., determination of *V*_max_ and *K*_m_ kinetic parameters) were analyzed by nonlinear regression of initial rates versus *M. luteus* substrate concentration. To assess inhibition, Tet009 was added to reactions at concentrations ranging from 0 to 200 ng·mL^−1^. Importantly, by using freeze‐dried *M. luteus* as a reporter substrate, we selectivity quantified hLYS‐mediated bacteriolysis independent of Tet009‐mediated membrane disruption and cell killing. All reactions were run in triplicate, and the maximum reaction velocity (*V*_max_) is reported as the hyperbolic asymptote and 95% confidence interval for the best‐fit regression line to a given data set.

## Results

Here we sought to evaluate antipseudomonal interactions between hLYS and six different AMPs, testing combinations against mucoid Xen05 and nonmucoid H1001 *P. aeruginosa* strains. Both Xen05 and H1001 have been engineered for bioluminescence via a genomically inserted *Photorhabdus luminescens* lux operon, and each has been validated as a tool for screening and evaluation of antibacterial agents, including biologics such as AMPs [19, 23, 24]. Importantly, the luminescence of these engineered strains has been shown to correlate with bacterial viability [20].

### Determination of single agent potency: EC_50_ assays

Our small panel of AMPs were derived from diverse origins (human, pig, bee, and purely synthetic), with two representatives each from the α‐helical, β‐sheet, and extended AMP structural classes (Table 1). We first analyzed dose–response potency of each AMP against both strains via a luminescence assay [19] (Fig. S1). Most AMPs exhibited similar EC_50_ values for the mucoid and nonmucoid strains (Table 1), and these values were largely consistent with previously published MIC values for each peptide [23, 24, 25, 26, 27, 28, 29, 30, 31, 32, 33].

**Table 1:** Antimicrobial agents used in this study.

Agent	Origin	Structure	Net charge	EC_50_ (μg·mL^−1^)		Sequence	Source	Reference
				Xen05	H1001			
hLYS	Human	Mixed	+5	990	890	UniProt—B2R4C5	Sigma Millipore (L1667)	[12]
LL37	Human	α helix	+6	5.0	6.1	LLGDFFRKSKEKIGKEFKRIVQRIKDFLRNLVPRTES	GenScript (> 95%)	[29]
hBD3	Human	β sheet	+11	6.0	7.0	GIINTLQKYYCRVRGGRCAVLSCLPKEEQIGKCSTRGRKCCRRKK	AnaSpec (AS‐60741)	[27]
PTG‐1	Pig	β sheet	+6	1.1	1.7	RGGRLCYCRRRFCVCVGR	Peptide 2.0 (> 95%)	[25]
HB71	Synthetic	Extended	+9	1.6	2.6	FAKKLAKKLKKLAKKLAK	Peptide 2.0 (> 95%)	[33]
Melittin	Bee	α helix	+5	4.5	3.5	GIGAVLKVLTTGLPALISWIKRKRQQ	GenScript (> 95%)	[31]
Tet009	Synthetic	Extended	+6	1.5	2.7	RRWKIVVIRWRR	GenScript (> 95%)	[24]

### Evaluation of antibacterial interactions: 2‐dimensional MIC or ‘checkerboard’ assays

Based on the EC_50_ values of AMP monotherapies, we designed checkerboard assays [21] wherein antibacterial interactions between hLYS and each AMP were quantified. Classical drug interactions such as synergy, additivity, or antagonism manifest characteristic trends across a given checkerboard assay plate, where FICI < 0.5 is defined as synergy, 0.5 ≤ FICI < 4 is defined as additivity, and FICI ≥ 4 is defined as antagonism [34]. Nonclassical interactions, as observed in some of our checkerboard results, might include a concentration‐dependent switch from synergy (or additivity) to antagonism. In extreme cases, we observed inversion of the dose–response curves for specific combinations and concentration ranges of our antibacterial agents, as discussed below.

**Fig. 1:**
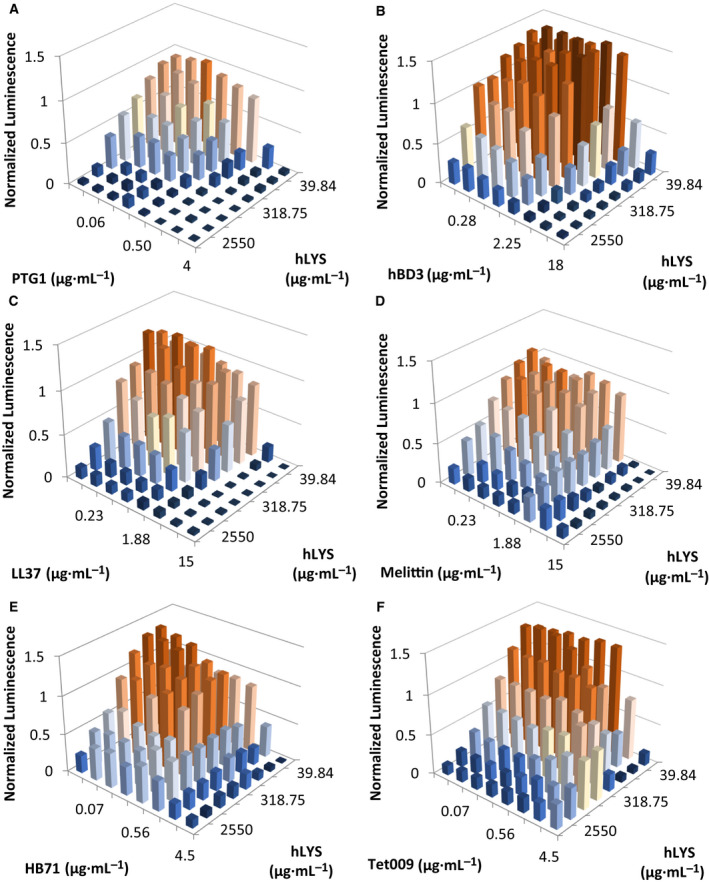
2‐D MIC assays (i.e., checkerboard assays) for hLYS and AMPs against mucoid *P. aeruginosa* strain Xen05. Peptides are (A) PTG1, (B) HBD3, (C) LL37, (D) melittin, (E) HB71, and (F) Tet009. Bacterial viability reported as luminescence units, and values are normalized to the luminescence of the no‐treatment control well. Antimicrobial therapies typically exhibit a dose–response killing curve from low to high concentrations, as seen for both PTG1 and hLYS in panel (A). In this study, noncanonical dose–response curves were observed for several hLYS‐AMP combinations, including (D) melittin, (E) HB71, and (F) Tet009. Shown in each panel are the mean values from three independent experiments.

**Fig. 2:**
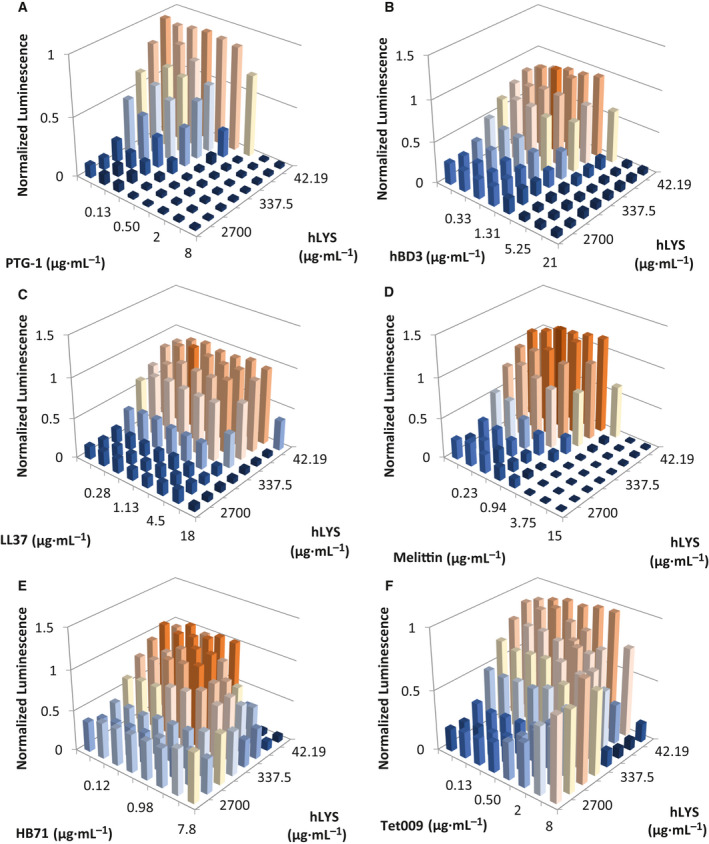
2‐D MIC assays (i.e., checkerboard assays) for hLYS and AMPs against nonmucoid *P. aeruginosa* strain H1001. Peptides are (A) PTG1, (B) HBD3, (C) LL37, (D) melittin, (E) HB71, and (F) Tet009. Bacterial viability is reported as luminescence units, and values are normalized to the luminescence of the no‐treatment control wells. Antimicrobial therapies typically exhibit a dose–response killing curve from low to high concentrations, as seen for both PTG1 and hLYS in panel (A). In this study, noncanonical dose–response curves were observed for several hLYS‐AMP combinations, including (E) HB71 and (F) Tet009. Shown in each panel are the mean values from three independent experiments.

Classical additive or synergistic activities were observed for combinations of hLYS with PTG1, HBD3, LL37, and melittin (Table 2), although the interactions were not always consistent between mucoid and nonmucoid strains. The porcine β‐sheet AMP PTG1 exhibited synergy with hLYS against both *P. aeruginosa* isolates, similar to prior literature reports (Figs 1A and 2A) [35]. The human β‐sheet AMP HBD3 exhibited additivity with hLYS against the mucoid strain Xen05 (Fig. 1B) and synergy with hLYS against the nonmucoid strain H1001 (Fig. 2B). The human α‐helical AMP LL37 exhibited additive hLYS interactions against both Xen05 and H1001 strains (Figs 1C and 2C), although FICI values were borderline synergistic (Table 2). Lastly, the α‐helical bee venom peptide melittin demonstrated hLYS synergy against the nonmucoid strain H1001 (Fig. 2D).

**Table 2:** FICI for AMP‐hLYS combinations. N.C., nonclassical interaction. See Figs 1 and 2.

AMP	FICI Xen05	FICI H1001
PTG‐1	0.3 ± 0.2	0.4 ± 0.1
HBD3	1.02 ± 0.09	0.3 ± 0.3
Melittin	N.C.	0.4 ± 0.2
LL37	0.5 ± 0.2	0.5 ± 0.2
HB71	N.C.	N.C.
Tet009	N.C.	N.C.

In the remaining hLYS‐AMP combinations, we observed nonclassical behavior of differing types. Against mucoid Xen05, the hLYS dose response at high melittin concentrations was inverted, showing greater luminescence at higher concentrations of hLYS (Fig. 1D). It should be noted, however, that all wells exhibited relatively low luminescence at high melittin concentrations, indicative of strong overall antibacterial activity. Against strain H1001, the extended synthetic peptide HB71 caused a similar inversion of hLYS dose response at high AMP concentrations, whereas the dose response for HB71 itself was largely flattened at intermediate luminescence values when combined with high hLYS concentrations (Fig. 2E). For mucoid strain Xen05, the hLYS dose response was flattened or even inverted at high HB71 concentrations, though similar to melittin combinations, the bacterial luminescence was generally low at high HB71 concentrations (Fig. 1E). Perhaps most striking, at high hLYS concentrations the Tet009 dose response was inverted with both strains (Figs 1F and 2F), where the effect was most pronounced with nonmucoid H1001. Similarly, we observed that the hLYS dose response against both strains was inverted at high Tet009 concentrations. As presented here, the various inverted dose–response curves are, to the best of our knowledge, the first description of nonclassical *in vitro* interactions between hLYS and AMPs. Although this panel of AMPs is too small to draw definitive conclusions, we noted that the AMP‐LYS interactions loosely correlated with AMP secondary structure: the two β‐sheet peptides generally exhibited hLYS synergy, whereas the two extended peptides generally manifested nonclassical antagonistic activity. Notably, our checkerboard results show that Tet009 and hLYS are mutually antagonistic, each inhibiting the other but only at higher concentrations.

### Analyzing Tet009 inhibition of hLYS catalytic activity

**Fig. 3:**
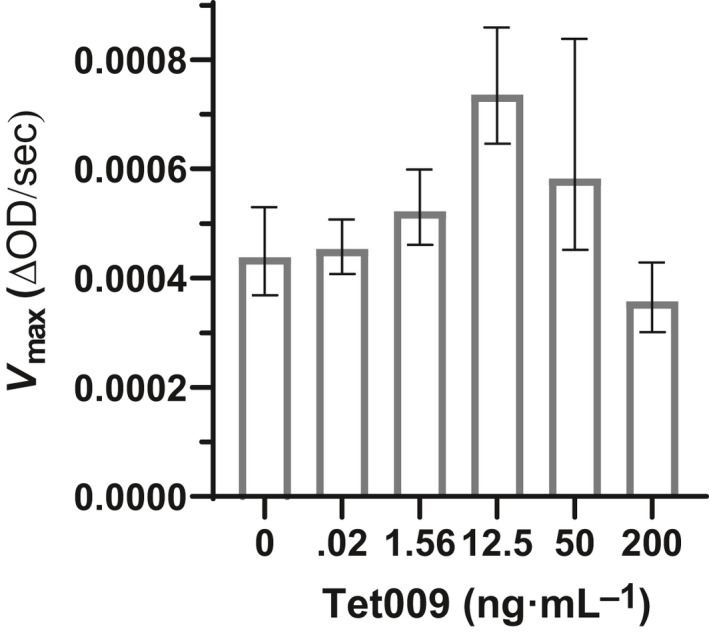
*V*_max_ values from pseudo‐Michaelis–Menten kinetic analysis of hLYS lytic rates. *V*_max_ values at different concentrations of Tet009 AMP reveal a nonclassical inhibitory effect manifested only at high Tet009 concentrations. All kinetic runs were evaluated in triplicate and shown are the means and 95% confidence intervals for *V*_max_, derived from the best curve fits. The hyperbolic curve fits for all reactions were compared, and it was found that the *V*_max_ values differed significantly among the data sets (*P* < 0.0001, extra sum of squares *F* test). Initial rates from linear regression of time course light scattering experiments at different substrate concentrations are shown in Fig. S2, as are best‐fit hyperbolas by nonlinear regression.

The strong dose–response inversion observed in the hLYS and Tet009 checkerboard assays suggested that Tet009 could be interfering with hLYS‐mediated peptidoglycan hydrolysis. To further probe this hypothesis, we employed a kinetic bacteriolysis assay using nonviable *M. luteus* cells as substrate [22]. Initial reaction rates were determined from slopes of the linear portions of time course data, and pseudo‐Michaelis–Menten kinetics were analyzed by nonlinear regression of initial rates versus *M. luteus* substrate concentration (Fig. S2). At Tet009 concentrations from 0.02 to 12.5 ng·mL^−1^, hLYS lytic rates (quantified as apparent *V*_max_) accelerated, reaching a maximum rate at 12.5 ng·mL^−1^ AMP (Fig. 3). However, the effect was reversed at Tet009 concentrations above 12.5 ng·mL^−1^, with the slowest apparent rate at 200 ng·mL^−1^ Tet009 (Fig. 3). While these experiments do not conclusively distinguish between direct Tet009 inhibition of hLYS catalysis and Tet009 competition for hLYS binding sites on *M. luteus* cell walls, the results do reinforce the observed nonclassical antagonism observed in the checkerboard assays. Specifically, we found complementary activity of the two agents up to a threshold Tet009 concentration followed by a switch to antagonism above that threshold.

## Discussion

To the best of our knowledge, these results represent the first observations of antagonism between hLYS and AMPs. Fully assessing the broader significance of these results, obtained in a screening medium, will require further study. However, we note that similar bioluminescence assays in DT buffer have been used previously for screening and identification of AMP therapeutic candidates [19, 24, 36]. Our results are therefore directly relevant to screening efforts aimed at identifying AMP‐enzyme combination therapies. Additionally, in a prior publication we made the unexpected observation that, in a murine model of acute *P. aeruginosa* lung infection, Tet009 and hLYS combination therapy manifested a weak trend toward reduced efficacy compared to hLYS monotherapy [37]. Thus, antagonistic interactions may indeed be relevant to *in vivo* environments. Notwithstanding the limitations of the *in vitro* methods employed here, our results advise that future development of lytic enzyme‐AMP combinations should carefully consider the potential for antagonism.

## Conflict of interest

KEG is a cofounder and member‐manager of Lyticon LLC, which holds a license for rights to engineered lysozyme biotherapies. No other authors have a conflict of interest. Potential conflicts of interest for KEG are under management at Dartmouth. The authors declare that the work presented here is free of any bias.

## Author contributions

KEG and IB designed the experiments. IB conducted the experiments. IB, LRD, CMB, and KEG analyzed the data. LRD, CMB, and KEG wrote the manuscript.

## Supporting information


**Fig. S1.** Dose–response curves of each antimicrobial agent against *P. aeruginosa* strains Xen05 (A‐F, M) and H1001 (G‐L, N). 95% confidence intervals for EC_50_ values in µg·mL^−1^: (A) 1.4–1.5; (B) 5.5–6.5; (C) 1.4–1.8; (D) 3.8–5.4; (E) 1.1–1.2; (F) 4.8–5.1; (G) 2.5–2.9; (H) 6.9–7.1; (J) 3.4–3.6; (K) 1.6–1.8; (L) 5.7–6.6; (M) 800–1200; (N) 700–1100. Experiments were conducted as technical triplicate or quadruplicate measurements and were repeated at least twice.

**Fig. S2.** Pseudo‐Michaelis–Menten kinetic analysis of hLYS lytic rates toward Gram‐positive *M. luteus* bacteria in the presence of the specified concentration of Tet009 AMP. All reactions were evaluated in triplicate and shown are the means and standard deviation of the initial rate at each bacterial substrate concentration. The best‐fit hyperbola for each data set is shown, and *V*_max_ values from the regressions are provided in Fig. 3.

**Table S1**. Literature Review of AMP Interactions with Lysozyme.

## Data Availability

All data will be made available by the corresponding author upon reasonable request.
