# Supplementary material for: Nonclassical antagonism between human lysozyme and AMPs against Pseudomonas aeruginosa
Source: FEBS Open Bio. 2021 Feb 5;11(3):705–13. doi: 10.1002/2211-5463.13094 (PMC7931236; doi:10.1002/2211-5463.13094)
Supplement: Supplementary file 1 — Fig. S1. Dose–response curves of each antimicrobial agent against P. aeruginosa strains Xen05 (A‐F, M) and H1001 (G‐L, N). 95% confidence intervals for EC50 values in µg·mL−1: (A) 1.4–1.5; (B) 5.5–6.5; (C) 1.4–1.8; (D) 3.8–5.4; (E) 1.1–1.2; (F) 4.8–5.1; (G) 2.5–2.9; (H) 6.9–7.1; (J) 3.4–3.6; (K) 1.6–1.8; (L) 5.7–6.6; (M) 800–1200; (N) 700–1100. Experiments were conducted as technical triplicate or quadruplicate measurements and were repeated at least twice. [file FEB4-11-705-s001.pdf]

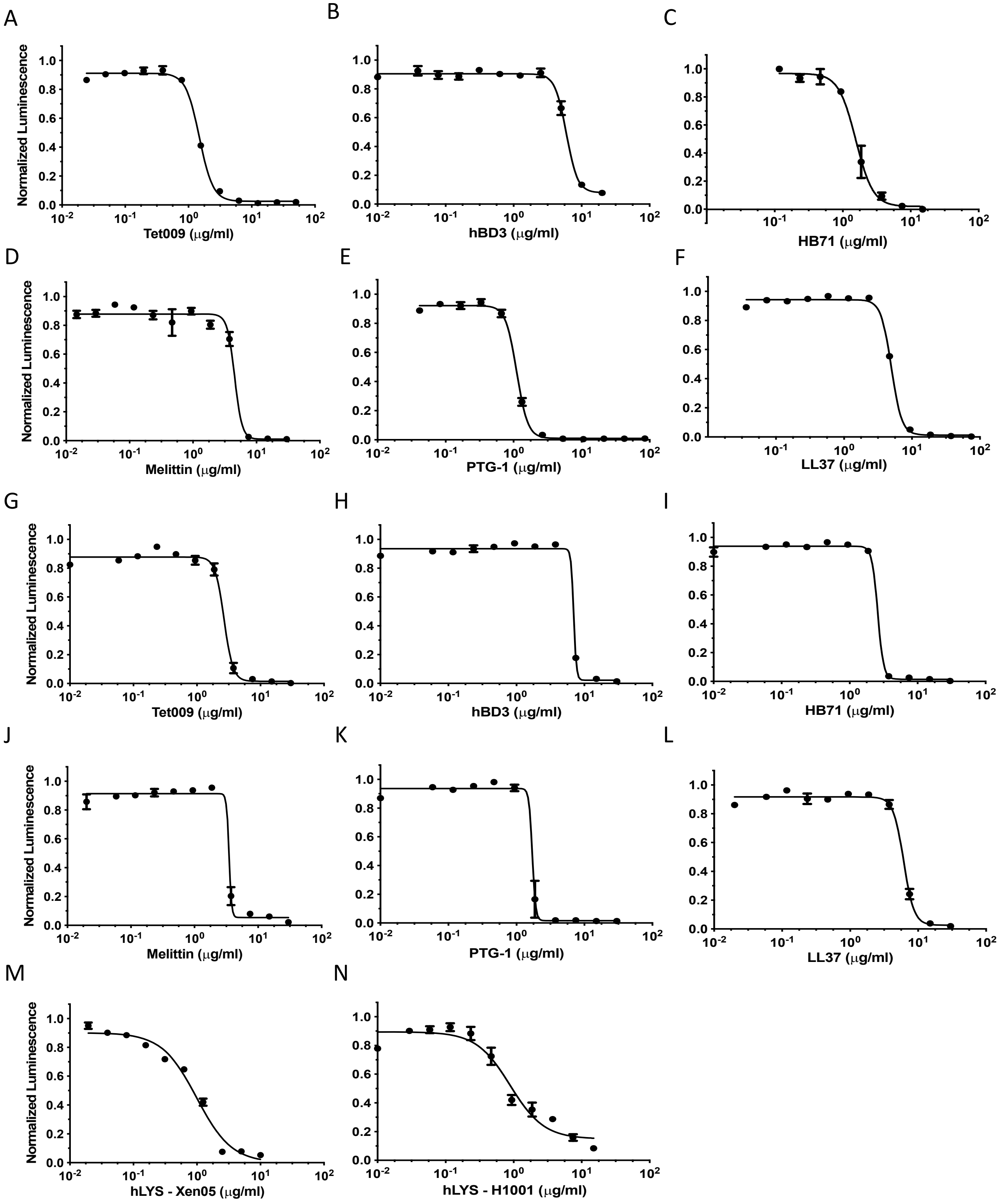

Fig. S1 - Dose response curves of each antimicrobial agent against P. aeruginosa strains Xen05 (A-F, M) and H1001 (G-L, N). 95% confidence intervals for EC50 values in µg/ml: (A) 1.4-1.5; (B) 5.5-6.5; (C) 1.4-1.8; (D) 3.8-5.4; (E) 1.1-1.2; (F) 4.8-5.1; (G) 2.5-2.9; (H) 6.9-7.1; (J) 3.4-3.6; (K) 1.6-1.8; (L) 5.7-6.6; (M) 800-1200; (N) 700-1100. Experiments were conducted as technical triplicate or quadruplicate measurements and were repeated at least twice.
