# Supplementary material for: Nonclassical antagonism between human lysozyme and AMPs against Pseudomonas aeruginosa
Source: FEBS Open Bio. 2021 Feb 5;11(3):705–13. doi: 10.1002/2211-5463.13094 (PMC7931236; doi:10.1002/2211-5463.13094)
Supplement: Supplementary file 2 — Fig. S2. Pseudo‐Michaelis–Menten kinetic analysis of hLYS lytic rates toward Gram‐positive M. luteus bacteria in the presence of the specified concentration of Tet009 AMP. All reactions were evaluated in triplicate and shown are the means and standard deviation of the initial rate at each bacterial substrate concentration. The best‐fit hyperbola for each data set is shown, and V max values from the regressions are provided in Fig. 3. [file FEB4-11-705-s002.pdf]

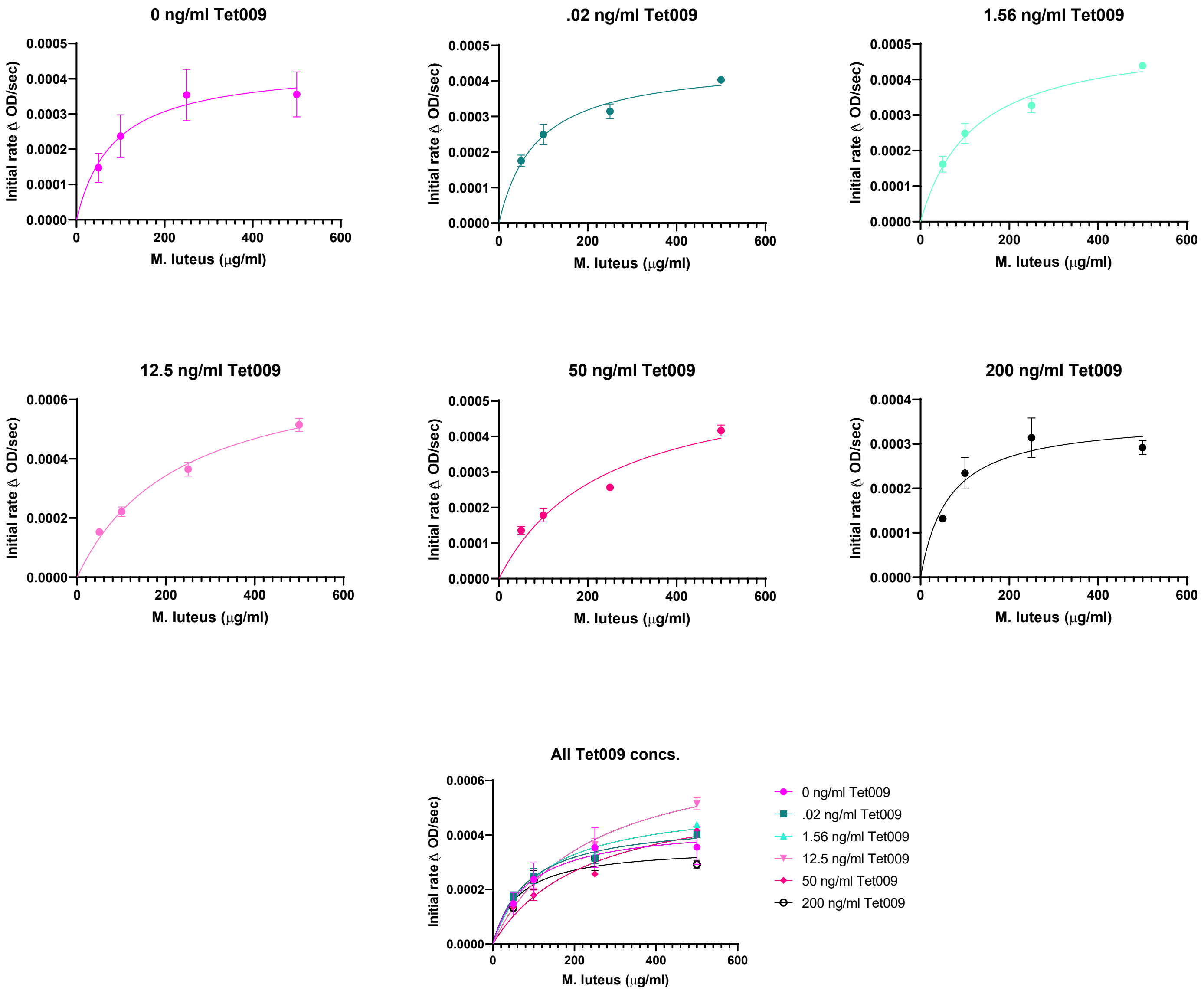

Fig. S2 - Pseudo-Michaelis-Menten kinetic analysis of hLYS lytic rates towards Gram-positive *M. luteus* bacteria in the presence of the specified concentration of Tet009 AMP. All reactions were evaluated in triplicate and shown are the means and standard deviation of the initial rate at each bacterial substrate concentration. The best fit hyperbola for each data set is shown, and V_{max} values from the regressions are provided in Fig. 3.
