# Supplementary material for: Nonclassical antagonism between human lysozyme and AMPs against Pseudomonas aeruginosa
Source: FEBS Open Bio. 2021 Feb 5;11(3):705–13. doi: 10.1002/2211-5463.13094 (PMC7931236; doi:10.1002/2211-5463.13094)
Supplement: Supplementary file 3 — Table S1. Literature Review of AMP Interactions with Lysozyme. [file FEB4-11-705-s003.pdf]

Table S1. Literature Review of AMP Interactions with Lysozyme

AMP	Interaction	Reference
AS-48	Synergy	(1) (2)
Bactenicin	Additive	(3)
GMAP2	Synergy	(4) (5)
hBD1	Synergy	(6)
	Additive	(7)
hBD2	Synergy	(6) (8)
	Additive	(7)
hBD3	Synergy	(6) (9) (10)
H-GADDDDD-OH	Additive	(11)
Indolicidin	Additive	(3)
Lactoferrin	Synergy	(7) (12) (13) (14)
	Additive	(15) (16)
Lactoferricin	Synergy	(17)
	Additive	(18)
LL37	Synergy	(6) (7) (10) (19) (20)
	Additive	(3)
Nisin	Synergy	(21) (16) (22)
OaBac5mini	Synergy	(16)
Parasin 1	Synergy	(23)
Polymyxin B	Synergy	(16)
PTG-1	Synergy/Additive	(3)
Puroindoline A+B	Synergy	(13)
SLPI	Synergy	(7)
SMAP29	Additive	(16)

Table S1 – References

1. Ananou S, Rivera S, Madrid MI, Maqueda M, Martínez-Bueno M, Valdivia E. 2018. Application of enterocin AS-48 as biopreservative in eggs and egg fractions: Synergism through lysozyme. LWT - Food Science and Technology 89:409-417.
2. Aguilar-Pérez C, Gracia B, Rodrigues L, Vitoria A, Cebrián R, Deboosère N, Song O-r, Brodin P, Maqueda M, Aínsa JA. 2018. Synergy between circular bacteriocin AS-48 and ethambutol against *Mycobacterium tuberculosis*. Antimicrobial Agents and Chemotherapy 62:e00359-18.
3. Yan H, Hancock REW. 2001. Synergistic Interactions between Mammalian Antimicrobial Defense Peptides. Antimicrobial Agents and Chemotherapy 45:1558-1560.
4. Sowa-Jasiłek A, Zydbicka-Barabas A, Staczek S, Wydrych J, Mak P, Jakubowicz T, Cytryńska M. 2014. Studies on the role of insect hemolymph polypeptides: *Galleria mellonella* anionic peptide 2 and lysozyme. Peptides 53:194-201.
5. Zydbicka-Barabas A, Mak P, Klys A, Skrzypiec K, Mendyk E, Fiołka MJ, Cytryńska M. 2012. Synergistic action of *Galleria mellonella* anionic peptide 2 and lysozyme against gram-negative bacteria. Biochimica et Biophysica Acta 1818:2623-2635.
6. Chen X, Niyonsaba F, Ushio H, Okuda D, Nagaoka I, Ikeda S, Okumura K, Ogawa H. 2005. Synergistic effect of antibacterial agents human beta-defensins, cathelicidin LL-37 and lysozyme against *Staphylococcus aureus* and *Escherichia coli*. Journal of Dermatological Science 40:123-132.
7. Singh PK, Tack BF, Jr PBM, Welsh MJ. 2000. Synergistic and additive killing by antimicrobial factors found in human airway surface liquid. American Journal of Physiology-Lung Cellular and Molecular Physiology 279:L799-L805.
8. Lee H-Y, Andalibi A, webster P, Moon S-K, Teufert K, Kang S-H, Li J-D, Nagura M, Ganz T, Lim DJ. 2004. Antimicrobial activity of innate immune molecules against *Streptococcus pneumonia, Moraxella catarrhalis* and nontypeable *Haemophilus influenzae*. BMC Infectious Diseases 4.
9. Maisetta G, Batoni G, Esin S, Luperini F, Pardini M, Bottai D, Florio W, Giuca MR, Gabriele M, Campa M. 2003. Activity of human beta-defensin 3 alone or combined with other antimicrobial agents against oral bacteria. Antimicrobial Agents and Chemotherapy 47:3349-3351.
10. Alaiwa MHA, Reznikov LR, Gansemer ND, Sheets KA, Horswill AR, Stoltz DA, Zabner J, Welsh MJ. 2014. pH modulates the activity and synergism of the airway surface liquid antimicrobials beta-defensin-3 and LL-37. PNAS 111:18703-18708.
11. Kalfa V, Brogden K. 1999. Anionic antimicrobial peptide-lysozyme interactions in innate pulmonary immunity. International Journal of Antimicrobial Agents:47-51.
12. André G, Politano W, Mirza S, Converso T, Ferraz L, Darrieux M. 2015. Combined effects of lactoferrin and lysozyme on *Streptococcus pneumoniae* killing. Microbial Pathogenesis 89:7-17.
13. Palumbo D, Iannaccone M, Porta A, Capparelli R. 2010. Experimental antibacterial therapy with puroindolines, lactoferrin and lysozyme in *Listeria monocytogenes* - infected mice. Microbes and Infection 12:538-545.
14. Leitch E, Willcox M. 1999. Elucidation of the antistaphylococcal action of lactoferrin and lysozyme. Journal of Medical Microbiology 48:867-871.
15. Ellison R, III, Giehl TJ. 1991. Killing of gram-negative bacteria by lactoferrin and lysozyme. Journal of Clinical Investigation 88:1080-1091.
16. Linden DSvd, Short D, Dittmann A, Yu P-L. 2009. Synergistic effects of ovine-derived cathelicidins and other antimicrobials against *Eschericia coli* O157:H7 and *Staphylococcus aureus* 1056 MRSA. Biotechnology Letters 31:1265-1267.
17. Facon MJ, Skura bJ. 1996. Antibacterial activity of lactoferricin, lysozyme and EDTA against *Salmonella enteritidis*. International Dairy Journal 6:303-313.
18. El-Sayed E, Mahfouz M, Abdelsalam A, Gawad IAE, Naim H. 2003. Antimicrobial activity of recombinant bovine lactoferrin and lactoferricin B and glycosylated human lysozyme. Milk Science International - Milchwissenschaft 58:266-270.
19. Bals R, Wang X, Zasloff M, Wilson JM. 1998. The peptide antibiotic LL-37/hCAP-18 is expressed in epithelia of the human lung where it has broad antimicrobial activity at the airway surface. PNAS 95:9541-9546.
20. Travis SM, Singh PK, Welsh MJ. 2001. Antimicrobial peptides and proteins in the innate defense of the airway surface. Current Opinion in Immunology 13:89-95.
21. Chung W, Hancock REW. 2000. Action of lysozyme and nisin mixtures against lactic acid bacteria. International Journal of Food Microbiology 60:25-32.
22. Sobrino-López A, Martín-Belloso O. 2008. Enhancing the lethal effect of high-intensity pulsed electric field in milk by antimicrobial compounds as combined hurdles. Journal of Dairy Science 91:1759-1768.
23. Zhao H, Tang J, Cao L, Jia G, Long D, Liu G, Chen X, Cai J, Shang H. 2015. Characterization of bioactive recombinant antimicrobial parasin I fused with human lysozyme expressed in the yeast *Pichia pastoris* system. Enzyme and Microbial Technology 77:61-67.
